# Validation of the Acute Physiology and Chronic Health Evaluation (APACHE) II and IV Score in COVID-19 Patients

**DOI:** 10.1155/2021/5443083

**Published:** 2021-06-19

**Authors:** Jeroen Vandenbrande, Laurens Verbrugge, Liesbeth Bruckers, Laurien Geebelen, Ester Geerts, Ina Callebaut, Ine Gruyters, Liesbeth Heremans, Jasperina Dubois, Bjorn Stessel

**Affiliations:** ^1^Department of Intensive Care and Anesthesiology, Jessa Hospital, Hasselt, Belgium; ^2^I-BioStat, Data Science Institute, Hasselt University, Martelarenlaan 42, Hasselt 3500, Belgium; ^3^UHasselt, LCRC, Agoralaan, Diepenbeek 3590, Belgium

## Abstract

**Background:**

Severity scoring systems are inherent to ICU practice for multiple purposes. Acute Physiology and Chronic Health Evaluation (APACHE) scoring systems are designed for ICU mortality prediction. This study aims to validate APACHE IV in COVID-19 patients admitted to the ICU.

**Methods:**

All COVID-19 patients admitted to the ICU between March 13, 2020, and October 17, 2020, were retrospectively analyzed. APACHE II and APACHE IV scores as well as SOFA scores were calculated within 24 hours after admission. Discrimination for mortality of all three scoring systems was assessed by receiver operating characteristic curves. Youden index was determined for the scoring system with the best discriminative performance. The Hosmer–Lemeshow goodness-of-fit test was used to assess calibration. All analyses were performed for both the overall population as in a subgroup treated with anti-Xa adjusted dosages of LMWHs.

**Results:**

116 patients were admitted to our ICU during the study period. 13 were excluded for various reasons, leaving 103 patients in the statistical analysis of the overall population. 57 patients were treated with anti-Xa adjusted prophylactic dosages of LMWH and were supplementary analyzed in a subgroup analysis. APACHE IV had the best discriminative power of the three scoring systems, both in the overall population (APACHE IV ROC AUC 0.67 vs. APACHE II ROC AUC 0.63) as in the subgroup (APACHE IV ROC AUC 0.82 vs. APACHE II ROC AUC 0.7). This model exhibits good calibration. Hosmer–Lemeshow *p* values for APACHE IV were 0.9234 for the overall population and 0.8017 for the subgroup. Calibration *p* values of the APACHE II score were 0.1394 and 0.6475 for the overall versus subgroup, respectively.

**Conclusions:**

APACHE IV provided the best discrimination and calibration of the considered scoring systems in critically ill COVID-19 patients, both in the overall group and in the subgroup with anti-Xa adjusted LMWH doses. Only in the subgroup analysis, discriminative abilities of APACHE IV were very good. This trial is registered with NCT04713852.

## 1. Background

Prognostication of critically ill patients using severity scores is inherent to intensive care unit (ICU) practice for a variety of reasons. Severity scores play a crucial role in comparing predicted versus observed outcomes, allowing evaluation of treatment and benchmarking of ICU performance. Severity scores are also useful in clinical research to facilitate comparison between control and study groups [1]. Therefore, prediction models must be subject to continuous quality control and repeated calibration [1].

The most commonly used ICU scoring system, Acute Physiology and Chronic Health Evaluation II (APACHE II), was designed in 1985 [2]. Over the last three decades, ICU practice has changed dramatically due to new knowledge, improved technology, and admission of more complex cases with multiple comorbidities. These changes in ICU practice have led to a discrepancy between the actual mortality and the estimated mortality, based on the APACHE II prediction model [1]. Continuous efforts to improve the accuracy of outcome prediction led to the development of the APACHE IV in 2006 [1]. In the US mixed ICU population, APACHE IV has shown excellent discriminative and calibration values [1]. In a mixed Indian ICU population, however, APACHE IV had better discriminative power than APACHE II, whilst both had poor calibration [3]. In studies by Lee and Brinkman, APACHE IV also revealed good discrimination but poor calibration [4, 5]. In a recent study by Ko et al. in a medical ICU population, APACHE IV did score good discrimination and calibration values, superior to APACHE II's discriminative and calibration power [6].

So far, little is known about the performance of ICU scoring systems in critically ill COVID-19 patients. Zou et al. analyzed discriminative abilities of APACHE II, Sequential Organ Failure Assessment (SOFA) score, and Confusion, Urea, Respiratory rate, Blood pressure, Age 65 (CURB65) score [7]. They found APACHE II to have superior discriminative power compared to SOFA and CURB65 [7]. In a letter to the editor [7], Stephens et al. noticed higher than expected mortality compared to relatively low APACHE II scores in Zou's study, raising questions about the calibration of APACHE II for COVID-19 patients [8].

More data on the association of APACHE score and COVID-19 ICU survival are available from a report of the U.K.'s Intensive Care National Audit and Research Centre (ICNARC) database [9]. In contrast to Zou's analysis, APACHE II appeared to be a poor discriminator for mortality [9]. However, the ICNARC data showed the same discrepancy between low APACHE scores associated with generally high mortality, again raising questions about the calibration of APACHE II score for critically ill COVID-19 patients.

In summary, APACHE scoring systems are not validated in critically ill COVID-19 patients. In our study, we aimed to validate the APACHE IV score as a discriminator for mortality in critically ill COVID-19 patients. Furthermore, we compared it with the discriminative power of APACHE II and SOFA on admission. Second, we aimed to determine cutoff points for mortality in the best discriminative scoring system. Last, we aimed to calibrate both APACHE score systems using the Hosmer–Lemeshow goodness-of-fit test.

## 2. Methods

This single-center, investigator-initiated, longitudinal, retrospective observational cohort study was performed at Jessa Hospital, Hasselt, Belgium. This study was approved by the Ethics Committee of Jessa Hospital, Hasselt, Belgium, on 4^th^ January 2021 and registered on clinicaltrials.gov (NCT04713852). Written informed consent was waived in light of the urgent need to collect data on the ongoing pandemic. This study is reported according to the STrengthening the Reporting of OBservational studies in Epidemiology (STROBE) statement [10].

### 2.1. Study Population

All adult patients diagnosed with COVID-19 pneumonia and admitted to the ICU from 13^th^ March 2020 until 17^th^ October 2020 were included in the study. Following the World Health Organisation (WHO) protocol [11], laboratory confirmation of COVID-19 infection was defined as a positive result on polymerase chain reaction (PCR) assays of nasopharyngeal swab samples or bronchoalveolar lavage. Only laboratory-confirmed patients were included in the analysis. From March 13^th^, 2020, until October 17^th^, 2020, data from 116 consecutive patients admitted to the ICU were prospectively entered into a customized database that included medical history, demographic data, clinical symptoms and signs, laboratory results, and clinical outcomes. This database was retrospectively reviewed. APACHE II and IV scores were calculated on admission to the ICU [1, 2]. The Sequential Organ Failure Assessment (SOFA) score was evaluated daily [12].

### 2.2. Standard Treatment Procedure

All COVID-19 patients were treated according to the COVID-19 protocol of the JESSA hospital, based on the latest insights on COVID-19 at that time point [13]. According to this protocol, all patients admitted to our ICU received an intravenous (IV) infusion with glucose 5% at 60 ml/h as maintenance fluid and stress ulcer prophylaxis with pantoprazole 40 mg IV daily. Prophylactic antibiotic therapy was initiated for five days, using amoxicillin-clavulanic acid 1 g intravenously four times a day or moxifloxacin 400 mg intravenously once daily in case of known allergy to penicillin. Ventilatory support was initiated with high-flow nasal cannula or noninvasive mechanical ventilation as long as the patient was cooperative with this treatment. In case of respiratory fatigue, patients were sedated and intubated, and invasive mechanical ventilation was started according to the ARDS-network guidelines. This was based on the first reports that viral pneumonia caused by SARS-CoV-2 mimicked an ARDS-like pattern [13]. Sedation was performed by a combination of propofol, midazolam, and piritramide aiming for the lowest level of sedation required to tolerate mechanical ventilation. Adjustments were guided by pulse oxymetry levels, which are continuously monitored, and arterial blood gasses took every four hours. In case of hypotension due to vasoplegia, norepinephrine was used as the first choice vasopressor.

### 2.3. Subgroup Analysis

All patients admitted to the ICU from March 13^th^, 2020, until March 30^th^, 2020, received routine low-dose pharmacological VTE prophylaxis, i.e., once-daily subcutaneous injection of nadroparin calcium 2850 IU, according to the current guidelines in critically ill patients [14, 15]. On March 30^th^, 2020, a high incidence of deep venous thrombosis was discovered [16], for which we changed our prophylactic anticoagulation protocol from prophylactic to intermediate dosages of low molecular weight heparin (LMWH) with plasma anti-Xa activity monitoring [17]. Anti-Xa activity was targeted at 0.3–0.5 IU/ml in patients without echographic findings of deep venous thrombosis (DVT) and 0.4–1 IU/ml in patients with screening duplex positive for DVT. Subgroup analysis was performed for patients admitted after March 30^th^, 2020, who were treated with the new LMWH dosing regimens. This subgroup is called the current treatment group.

### 2.4. Outcome Parameter

The primary endpoint was ICU mortality. Hence, the study population was divided into participants who had died during their ICU stay and participants who were discharged from the ICU alive. Secondary outcomes included the incidence of VTE, acute kidney injury, and continuous renal replacement therapy (CRRT), length of stay (LOS) in the ICU and hospital LOS, and highest bilirubin, AST, and ALT during ICU stay. The dataset was closed on November 10^th^, 2020.

### 2.5. ICU Scoring Systems

APACHE II, APACHE IV, and SOFA scores were calculated via mdcalc.com within the first 24 hours after admission to our ICU. The data showing the highest patient severity were extracted from the ICU patient record.

### 2.6. Statistical Analysis

For descriptive purposes, continuous data are shown as mean ± standard deviation (SD) and categorical data are presented as frequencies (%).

The performances of the three ICU scoring systems were evaluated by means of logistic regression models with ICU mortality as the dependent variable and each time one of the scoring systems as an independent variable. The area under the receiver operating characteristic (AUC ROC) curve was used to describe the discriminative power for ICU survivors and nonsurvivors using APACHE II and IV versus SOFA-on-admission scores. The predictive value is classified based on the AUC as excellent discrimination (AUC value 0.99–0.9), very good (0.89–0.8), good (0.79–0.7), moderate (0.69–0.6), or poor (<0.6) [3]. The nonparametric approach of DeLong et al. was used to compare two ROC curves [18]. Calibration was assessed using the Hosmer–Lemeshow goodness-of-fit test and a calibration plot. The calibration plot shows predicted mortality probabilities versus observed probabilities. The measures of discrimination and calibration were calculated using 10-fold cross-validation.

Cutoff values for the best scoring system were determined based on the maximum Youden index (*J* = sensitivity + specificity − 1). Based on the cutoff value, patients were divided into two groups, and their survival curves were compared using Kaplan–Meier analysis.

All analyses were performed both in the total cohort (103 patients) as well as in the subgroup (current treatment group).

A 5% level of significance was used; no correction for multiple testing was used. All analyses were conducted with SAS software, version 9.4 of the SAS System for Windows.

## 3. Results

**Figure 1:**
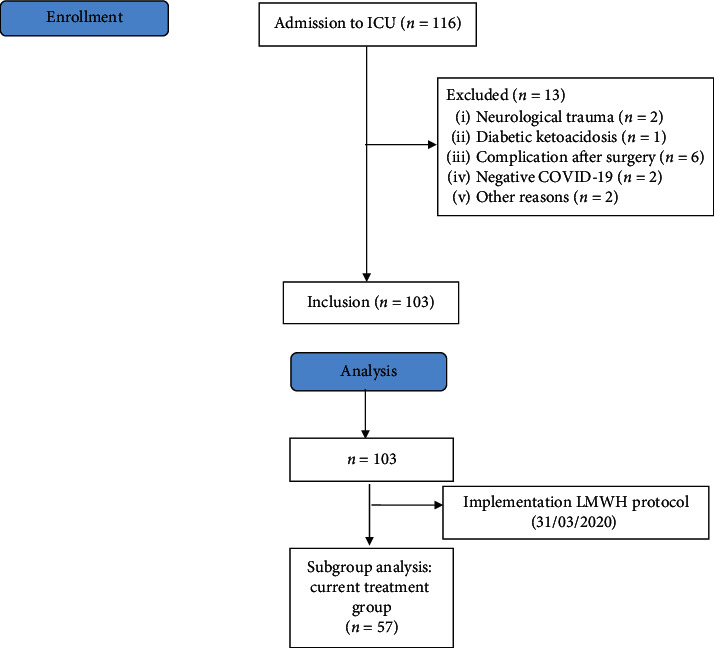
Study flowchart.

STROBE flowchart depicting inclusion and exclusion is shown in Figure 1. A total of 116 patients were admitted to the ICU between March 13^th^ and October 17^th^, 2020. 13 patients were excluded from further analysis: six patients were admitted for monitoring after major surgery, one diabetic patient was admitted in ketoacidosis, two were excluded because their prognosis was impaired by cerebral trauma, two were excluded due to a negative COVID-19 test, and two were excluded for other reasons. Of the remaining 103 patients, 57 (55, 3%) were treated with the intensified thromboprophylaxis protocol for whom a subanalysis was performed.

Demographic data are presented in Table 1.

Sixty-three (61.2%) patients were male. The mean age of the patients was 68.2 (±11.31) years. The mean APACHE II score was 12.49 (±4.94), whereas the mean APACHE IV score was 46.21 (±15.24). SOFA score on admission was 4.52 (±2.99). These scores are slightly lower for the current treatment group.

Out of 103 patients, 30 (29.1%) died during ICU stay, of which 10 were in the anti-Xa guided LMWH group (Table 2).

### 3.1. Discriminative Value of the Scoring Systems

Ten-fold cross-validation ROC AUC values are given in Table 3. For the overall group, APACHE IV (AUC 0.67) had a higher area under the curve values than SOFA scores on admission (0.53) (*p*=0.03). An AUC of 0.67 and an AUC of 0.63 (APACHE II) reflect moderate discriminative power for APACHE IV and II, whereas the SOFA score on admission had poor discriminative power in the total cohort.

**Figure 2:**
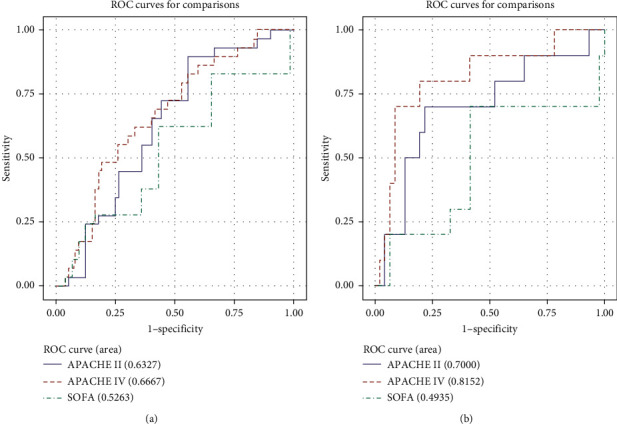
Ten-fold cross-validated ROC curves for the overall group (a) and current treatment group (b).

Analyses of ROC curves in the subgroup showed that APACHE IV (AUC 0.82) had very good discriminative abilities. We found good discrimination for APACHE II (AUC 0.7) and poor discriminative power for SOFA scores on admission (AUC 0.49). Receiver operator curves of cross-validated data are shown in Figure 2 (2(a), total group and 2(b), current treatment group).

**Figure 3:**
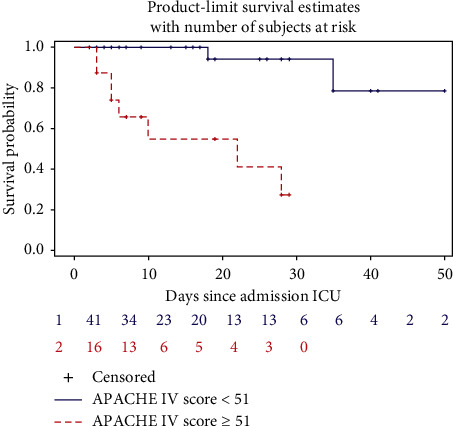
Kaplan–Meier survival curve of critically ill COVID-19 patients stratified by the APACHE IV score. The number of patients at risk is shown in the graph.

The Youden index for the best discriminative scoring system, being the APACHE IV in the subgroup, was determined next. The maximal Youden index under current treatment standards was 0.629, corresponding to an APACHE IV score cutoff point of 51. Patients with APACHE IV score > 51 showed significantly higher mortality (8/16, 50%) than patients with APACHE IV score < 51 (*p* < 0.0001 by the log-rank test). In the latter group, two patients out of 41 (4.88%) deceased. These findings are represented in a Kaplan–Meier survival curve (Figure 3).

### 3.2. Calibration

Results for calibration are displayed in Table 3.

The power of the HL statistic to detect badly calibrated models is low in small datasets. But based on this statistic, all scoring systems seem to be well-calibrated (*p* > 0.05). In both groups, APACHE IV had the highest efficacy for calibration with the Hosmer–Lemeshow *p* value of 0.9234 for the overall population and 0.8017 in the subgroup.

**Figure 4:**
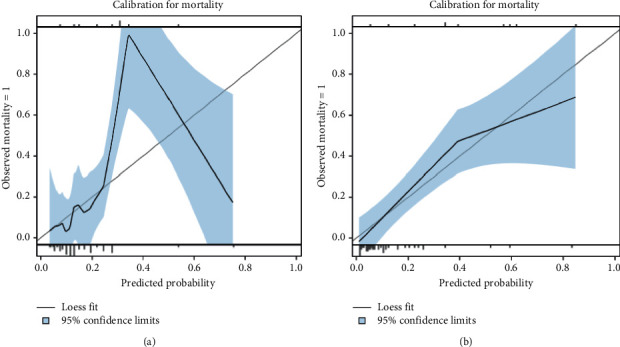
Calibration plots for mortality of subgroup analysis for APACHE II (a) and APACHE IV (b).

Calibration plots, however, show very wide 95% CIs (Figure 4). APACHE II (HL *p*=0.6475) shows an underestimation of mortality at lower to middle percentiles in the subgroup analysis (Figure 4(a)). APACHE IV calibration plot for the subgroup (HL *p*=0.802) shows a tendency of underestimation of mortality at lower percentiles and overestimation of mortality at higher percentiles at risk (Figure 4(b)).

## 4. Discussion

In this cohort study of ICU patients with severe SARS-CoV-2 pneumonia, the following important observations on the predictive value of APACHE II and IV scores versus SOFA scores on admission for ICU mortality were made. Both in the total cohort and in the subgroup, APACHE IV scores on admission were superior to SOFA scores on admission in predicting ICU mortality. After correcting for optimism, discriminative abilities of APACHE IV scores on admission were still very good (AUC 0.82) in the subgroup but only moderate (AUC 0.67) in the total cohort. Finally, the APACHE IV severity scoring system also marks the highest calibration values compared to APACHE II and SOFA. However, the APACHE IV calibration plot for the subgroup also shows an ambivalent risk prediction, i.e., an underestimation of mortality at lower percentiles and a distinct overestimation of mortality at higher percentiles at risk.

Critical care severity scores are important for quality reasons and benchmarking but might also be important for individual patient decision-making in case of ICU bed shortage. However, none of the existing scoring systems have been validated in critically ill COVID-19 patients. Also, up to now, no study has tried to validate APACHE IV in COVID-19 patients, although APACHE IV is expected to perform better in disease-specific subgroups [1]. A study by Zou et al. at the beginning of the pandemic showed excellent discriminative power for the APACHE II score (AUC 0.966) [7]. Zou et al., however, did not calibrate their model. Their primary goal was to investigate the APACHE score as a predictor for survival to facilitate end-of-life decision-making at the very beginning of the pandemic. Therefore, their study included patients admitted in Wuhan between January 10^th^, 2020, and February 10^th^, 2020 [7]. In the meantime, new insights into the COVID-19 have led to a different treatment strategy such as the use of corticosteroids (RECOVERY) [19] and intermediate doses of LMWHs [17, 20] in the critically ill. Therefore, in this retrospective study, we discriminated between the total population and subgroup analysis in patients admitted as of March 2020, who were treated with anti-Xa adjusted elevated dosages of LMWHs immediately after ICU admission. SOFA scores on admission were found to be a poor discriminator for mortality, in both the overall group and the subgroup. This might not be surprising since SOFA scores are designed as a follow-up scoring system rather than a relevant severity score on admission. APACHE scores are by definition scoring systems within the first 24 hours after ICU admission and are developed to estimate ICU mortality. We found the APACHE II score to have moderate discriminative abilities in the subgroup as well as in the overall group. The latter is in contrast to Zou's study, where APACHE II ROC AUC showed excellent discrimination by the APACHE II score at the beginning of the pandemic [7]. One possible explanation might be that the mean APACHE II score in our cohort was lower and more compressed (12.49 ± 4.94) compared to that in Zou's population (mean APACHE II 15.05 ± 7.71), making it more difficult to discriminate between higher and lower scores. We found the best ROC AUCs for the APACHE IV scoring system demonstrating good discrimination in the overall group and very good in the current treatment group, which is closest to current therapy guidelines.

Calibration by means of Hosmer–Lemeshow goodness-of-fit statistics showed all scoring systems to be effective in this study. APACHE IV showed the best efficacy in both the overall group and in the subgroup. These calibration insights are novel since Zou et al. did not validate for calibration, and up until now, no other reports have been published in the literature [7]. In a letter to the editor, Stephens et al. did raise questions about calibration of APACHE II for COVID-19 patients since both in the UK's database and in Zou's cohort, relatively high mortality rates for lower values of APACHE II were noted [8]. Our calibration plot confirmed this finding of underestimation of actual mortality in the lower to middle percentiles at risk in the APACHE II scoring system. In our study, APACHE IV showed superior calibration compared to SOFA. Calibration of APACHE IV scores was first performed by Zimmerman in a US mixed ICU population [1]. However, Varghese and Lee were unable to calibrate APACHE IV in an Indian and a Korean ICU population, respectively [3, 4]. Brinkman was not able to calibrate APACHE IV in a Dutch single-center ICU either [5]. Ko et al., on the other hand, demonstrated superior effectiveness of APACHE IV scores compared to APACHE II scores [6]. Interestingly, in Ko's study, 46.6% of the patients were admitted with respiratory failure, which is the primary reason for ICU admission in COVID-19 patients as well [6]. Indeed, conflicting results in calibration values can be explained by differences in study populations and treatment regimens [21].

Limitations of this study include the retrospective single-center design with low numbers of patients compared to the original APACHE validation studies. We therefore performed a model optimism correction by cross-validation of our data. Another pitfall is the quickly augmenting ICU knowledge about COVID-19, which leads to frequent changes in therapeutic strategies, creating a heterogeneous patient population. This accounts definitely for COVID-19. Therefore, one validation study might not resemble others performed during a different timespan. This statement is confirmed in this study. The results of the overall analysis and subanalysis in patients treated according to current guidelines are not resemblant.

## 5. Conclusion

In summary, we found very good discrimination by ROC AUC and satisfying calibration for APACHE IV score in critically ill COVID-19 patients under the current treatment strategy. APACHE IV performed better than SOFA both for discrimination and for calibration in our population. Both the insights in APACHE IV and in calibration are novelties in COVID-19 literature. The downside of this analysis is the limited number of patients. Larger, multicenter trials in different ethnic groups are needed to further validate APACHE IV for COVID-19 patients.

## Figures and Tables

**Table 1:** Baseline demographics.

	COVID-19 ICU patients (*n* = 103)	Current treatment group (LMWH protocol) (*n* = 57)
Demographics		
Age (years)	68.20 ± 11.32	67.63 ± 12.65
Gender (male/female)	46/57	30/27
BMI (kg/m^2^)	28.03 ± 5.19	28.41 ± 5.20

Comorbidities		
Smoking	5 (4.9%)	0 (0.0%)
Arterial hypertension	62 (60.2%)	33 (57.9%)
Diabetes	33 (32.0%)	18 (31.6%)
Respiratory disease	20 (19.4%)	8 (14.0%)
Malignancy	11 (10.7%)	7 (12.3%)
Chronic kidney disease	15 (14.6%)	9 (15.8%)
Chronic liver disease	3 (2.9%)	2 (3.5%)
Cardiovascular disease	39 (37.9%)	27 (47.4%)
Chronic bowel disease	8 (7.8%)	4 (7.0%)
Chronic nervous disease	1 (1.0%)	1 (1.8%)
Cerebrovascular disease	18 (17.5%)	12 (21.1%)
Haematological disease	9 (8.7%)	5 (8.8%)
Rheumatological disease	8 (7.8%)	4 (7.0%)
Dementia	2 (1.9%)	1 (1.8%)
HIV/AIDS	0 (0.0%)	0 (0.0%)

Clinical data on admission		
Fever	77 (74.8%)	36 (63.2%)
Cough	82 (80.4%)	39 (69.6%)
Dyspnoea	75 (73.5%)	38 (67.9%)
Sputum production	11 (10.8%)	6 (10.7%)
Myalgia	52 (51.0%)	28 (50.0%)
Headache	10 (9.8%)	4 (7.1%)
Diarrhoea	19 (18.6%)	12 (21.4%)
Respiratory rate	25.11 ± 6.66	26.00 ± 7.41
Systolic blood pressure	142.91 ± 26.70	144.72 ± 25.97
Diastolic blood pressure	90.73 ± 17.12	88.39 ± 14.29
Heart rate	88.44 ± 19.79	
Urine output day 1	1275.21 ± 822.00	1282.35 ± 726.99
GCS (total)	14.22 ± 1.33	14.52 ± 1.16
P/F ratio	117.44 ± 78.95	124.65 ± 61.48

Laboratory findings		
Red blood cell count (10^12^/l)	4.09 ± 0.74	3.84 ± 0.74
White blood cell count (10^9^/l)	8.77 ± 5.08	10.89 ± 7.63
Lymphocytes (%)	19.91 ± 36.28	29.45 ± 47.90
Platelets (10^9^/l)	246.15 ± 101.72	239.64 ± 100.24
Bilirubin (mg/l)	0.66 ± 0.59	0.72 ± 0.76

Length of stay (before ICU) (days)	3.10 ± 5.12	3.95 ± 6.27
SOFA (on ICU admission)	4.52 ± 2.99	4.18 ± 2.51
APACHE II (on ICU admission)	12.49 ± 4.94	11.96 ± 6.10
APACHE IV (on ICU admission)	46.21 ± 15.24	42.61 ± 14.73
Clinical frailty index	4.01 ± 1.58	3.91 ± 1.65

Results are displayed in mean ± SD or as numbers (%).

**Table 2:** Therapy and outcomes.

	COVID-19 ICU patients (*n* = 103)	Current treatment group (LMWH protocol) (*n* = 57)
Therapy		
Mean daily LMWH dose		
Nadroparin (ml)	1.00 ± 0.23	1.04 ± 0.24
Nadroparin (IU)	9500 ± 2185	9880 ± 2280
Antiviral treatment	6 (5.8%)	5 (8.8%)
Antibiotic treatment	95 (92.2%)	50 (87.8%)
Antifungal treatment	9 (8.7%)	6 (10.5%)
CRRT	18 (17.6%)	4 (7.1%)
ECMO	4 (7.0%)	4 (3.9%)
Oxygen treatment	101 (98.1%)	55 (96.5%)
Vasopressors	56 (54.3%)	21 (38.9%)
Neuromuscular blockers	47 (47.5%)	16 (30.2%)
Prone position	47 (46.1%)	24 (42.9%)
Corticosteroids	46 (44.7%)	32 (56.1%)

Outcomes		
Length of stay ICU	8.47 ± 9.8	10.30 ± 1.53
Length of stay hospital	30.26 ± 24.3	30.78 ± 3.2
GFR	74.52 ± 28.59	75.61 ± 30.94
Acute kidney failure	45 (43.7%)	16 (28.1%)
ICU mortality	30 (29.1%)	10 (17.5%)

Results are displayed in mean ± SD or as numbers (%).

**Table 3:** Results of the univariate logistic regression model.

Severity score	*p* value	OR (95% CI)	AUC	HL statistic (*p* value)
Total group				
APACHE II	0.0351	1.10 (1.01, 1.21)	0.63	10.98 (0.1394)
APACHE IV	0.0042	1.05 (1.02, 1.08)	0.67	2.54 (0.9234)
SOFA on admission	0.1501	1.11 (0.96, 1.27)	0.53	10.06 (0.0736)
Comparison of AUC ROC				
APACHE IV versus APACHE II	0.4835			
APACHE IV versus SOFA	0.0389			
APACHE II versus SOFA	0.1181			

Current treatment group				
APACHE II	0.0260	1.17 (1.02, 1.34)	0.70	5.10 (0.6475)
APACHE IV	0.0018	1.11 (1.04, 1.18)	0.82	3.06 (0.8017)
SOFA on admission	0.1464	1.20 (0.94, 1.52)	0.49	9.97 (0.0760)
Comparison of AUC ROC				
APACHE IV versus APACHE II	0.1477			
APACHE IV versus SOFA	0.0047			
APACHE II versus SOFA	0.1271			

The statistical significance of the severity score, the odds ratio (OR) and 95% confidence interval, the AUC values, and the Hosmer–Lemeshow (HL) test statistics are displayed for the overall group and current treatment group.

## Data Availability

Due to the applicable privacy regulation (GDPR) and good clinical practices (GCP) legislation, the full underlying dataset supporting the study cannot be provided. This dataset contains potentially identifying information, for example, age, BMI, and comorbidities such as diabetes leading to a unique subject in the dataset. Therefore, descriptive statistics have been used for a general overview of our study population, and all other relevant information is provided in Table 1. Anonymized data are available upon motivated request to Prof. Dr. Björn Stessel (bjorn.stessel@jessazh.be) and Jessa Ziekenhuis (dpo@jessazh.be).

## Abbreviations

ICU: Intensive care unit
APACHE: Acute Physiology and Chronic Health Evaluation
COVID-19: Coronavirus disease 2019
SOFA: Sequential organ failure assessment
LMWH: Low molecular weight heparin
ROC: Receiver operating characteristic
AUC: Area under curve
ICNARC: U.K.'s Intensive Care National Audit and Research Centre
STROBE: Strengthening the reporting of observational studies in epidemiology
PCR: Polymerase chain reaction
ARDS: Acute respiratory distress disease
VTE: Venous thromboembolism
DVT: Deep venous thrombosis
CRRT: Continuous renal replacement therapy
LOS: Length of stay
SD: Standard deviation.
